# Investigating the Genetic Association of Selected Candidate Loci with Alopecia Areata Susceptibility in Jordanian Patients

**DOI:** 10.3390/medicina61030409

**Published:** 2025-02-26

**Authors:** Laith N. AL-Eitan, Maryam K. Alasmar, Hanan A. Aljamal, Ahmad H. Mihyar, Mansour A. Alghamdi

**Affiliations:** 1 Department of Biotechnology and Genetic Engineering, Jordan University of Science and Technology, Irbid 22110, Jordan; 2 Department of Anatomy, College of Medicine, King Khalid University, Abha 62529, Saudi Arabia; m.alghamdi@kku.edu.sa; 3 Genomics and Personalized Medicine Unit, The Center for Medical and Health Research, King Khalid University, Abha 62529, Saudi Arabia

**Keywords:** skin disease, autoimmunity, genetic association, HLA genes, alopecia

## Abstract

*Background and Objectives*: Alopecia areata (AA) is a common cell-mediated autoimmune disease of the hair follicle that results in hair loss patches, affecting males and females of all ages and ethnicities. Although its etiology is not fully understood, AA is hypothesized to have a multifactorial basis with a strong genetic association. This study aims to replicate the genetic association of several risk loci in the Jordanian population for the first time. *Materials and Methods*: Genomic DNA samples of 152 patients with AA and 150 control individuals were extracted from EDTA blood tubes collected from dermatology clinics, in addition to the clinical data of participants. Genetic sequencing of the 21 targeted risk loci was carried out using the Sequenom MassARRAY^®^ system (iPLEX GOLD), and the results were statistically analyzed using the Statistical Package for the Social Sciences. *Results*: The results compared the distribution of alleles and genotypes and the association between control individuals and AA patients. However, our results do not support a significant association of all of the 21 SNPs in our AA cohort (*p* > 0.05). *Conclusions:* Our data emphasize that AA has a varied genetic component between ethnic groups and suggest that other additional environmental and psychological triggers may be involved.

## 1. Introduction

Alopecia areata (AA) is one of the most prevalent skin diseases characterized by non-scarring inflammatory hair loss [1,2,3]. The lifetime incidence of AA varies among populations: according to recent studies in the US and Saudi Arabia, the prevalence values of the disease are 0.09% and 2.3%, respectively [4,5]. The disease is generally considered an aberrant T-cell-mediated autoimmune response that attacks anagen hair follicles, resulting in the collapse of their immune privilege [6,7,8]. Hair loss in AA can manifest in many forms, typically beginning with well-defined bald patches that can progress into severe forms and may spread to affect the entire scalp (alopecia totalis; AT) or the entire body (alopecia universalis; AU) [9,10]. Furthermore, nail changes are common among AA patients, in addition to abnormalities of the sebaceous and sweat glands [11,12]. Given these clinical manifestations, AA can adversely impact patients’ quality of life and psychological well-being [13].

Alopecia areata (AA) is a multifactorial disease with a complex genetic inheritance pattern, as demonstrated by several association studies utilizing whole-genome, whole-exome, and family-based approaches. The estimated heritability of AA risk in first-degree relatives ranges from 5% to 8% [9,14,15,16]. Both environmental and genetic factors are believed to be essential in disease development. The variants of genes regulating the immune response, including both human leukocyte antigen (HLA) genes and non-HLA genes, have been identified as critical components in AA susceptibility [17,18]. While the identification of susceptibility loci has advanced our understanding of the genetic basis of AA, the disease prognosis remains unpredictable and highly variable, and its underlying etiopathogenesis is still not yet fully elucidated.

Given the observed variation in the genetic basis of alopecia areata (AA) across different populations, this study aims to investigate the genetic association of 21 candidate loci potentially contributing to AA susceptibility in the Jordanian population for the first time. The selected loci were chosen based on findings from prior studies conducted in other populations, yet they have not been previously explored in a Jordanian cohort [2,3,14,19,20,21,22,23,24,25,26,27,28,29,30,31,32,33,34]. By assessing the relationship between these polymorphisms and AA development, this study seeks to offer new insights into the genetic factors influencing the disease in this population. Furthermore, the results of this study could aid in advancing personalized medicine approaches and pharmacogenomics for more effective treatments of AA.

## 2. Materials and Methods

### 2.1. Study Population and Sample Source

This study was approved by the Institutional Review Board (IRB) at the Jordan University of Science and Technology (JUST). In compliance with the Declaration of Helsinki, written informed consent was obtained from all 302 study participants. Blood samples and clinical data were collected from 152 patients with AA and 150 ethnically matched control individuals. All methods were conducted in accordance with the relevant guidelines and regulations. Patients were recruited from the Dermatology, Venereology, and Laser Department that was established by the Jordanian Royal Medical Services (JRMS) in 1974, starting at King Hussein Medical City, Amman, Jordan, and were assessed based on the alopecia investigational assessment guidelines [18]. The inclusion criteria for this group ensured that all participants must be diagnosed with alopecia areata from the general dermatology clinic according to the alopecia investigational assessment guidelines [18]. The exclusion criteria for this group excluded any participants who were not diagnosed as AA patients. The participants in the control group were from dermatology clinics at King Abdullah University Hospital (KAUH), Irbid, Jordan. The study included individuals who were generally healthy and had no first-degree relatives with autoimmune diseases. Participants with any autoimmune disorders were excluded. Additional exclusion criteria included individuals with a recent history of debilitating diseases, whether acute or chronic, as well as scalp or skin conditions, such as psoriasis or seborrheic dermatitis, which could potentially confound the results. The sample collection for this study was conducted over three months from June to September 2017, following the ethical approval obtained on 6 April 2017. Participants were carefully chosen from the same geographic region and ethnic background to minimize genetic heterogeneity within the study cohort, thereby reducing confounding effects caused by population stratification.

### 2.2. SNP Selection

The gene and SNP selection for the current study was based on previously published results. The genes and SNPs were correlated with either AA susceptibility or dermatological characteristics related to AA diagnosis, like hair loss or autoimmune disease susceptibility. Global databases were used to further confirm the gene and SNP selections made in this study [35,36].

This genetic association case–control study for AA is the first of its kind to target 21 single-nucleotide polymorphisms (SNPs) within the following candidate genes among Jordanians: *PMS2* (rs1805323) [19], *PRDX5* (rs574087) [20], *ATXN2* (rs653178) [14], *MIF* (rs755622) [3], *LTF* (rs1126477) [21], *HFE* (rs1799945) [22], *HLA-DMB* (rs2071555) [23], *CD96* (rs2276872), *DMBT1* (rs2277244) [24], *ERBB3* (rs2292239) [25], *CHIT1* (rs2297950) [26], *PTPN22* (rs2476601) [2], *DEFB1* (rs2738047) [27], *ACOXL* (rs3789129) [14], *CLEC16A* (rs3862469) [28], *C20orf185* (rs4911290) [29], *MICA* (rs9380254) [30], *RAET1L* (rs9479478) [31], *CIITA* (rs78108426) [32], *HLA-A* (rs60304108) [33], and *GBP4* (rs17130745) [34].

### 2.3. DNA Extraction and Genotyping 

Genomic DNA (gDNA) was isolated from ethylenediaminetetraacetic acid (EDTA)-treated peripheral blood using a Wizard^®^ Genomic DNA Purification Kit (Qiagen, Hilden, Germany), which was provided for use in this study as part of a research collaboration [37]. Sequencing was carried out at the Australian Genome Research Facility (AGRF) using the Sequenom MassARRAY^®^ system (iPLEX GOLD) (San Diego, CA, USA).

### 2.4. The Sequenom MassARRAY^®^ System (iPLEX GOLD)

Samples passing quality control assessments were genotyped at the Australian Genome Research Facility using the Sequenom MassARRAY^®^ system (iPLEX GOLD). This high-throughput, cost-effective platform allows for genotyping hundreds to thousands of samples for tens to hundreds of SNPs. It combines single-base primer extension and MALDI-TOF mass spectrometry for precise SNP genotyping, supporting up to 40 SNPs in 384-well plates with automated barcoding for tracking.

### 2.5. SNP Analysis Using Sequenom MassARRAY Genotyping

The process begins with multiplex PCR to amplify regions with target SNPs, producing short PCR products. Residual dNTPs are removed with shrimp alkaline phosphatase (SAP) and the MassEXTEND^®^ primer extension process to create allele-specific DNA products. Products are analyzed via MALDI-TOF mass spectrometry, where time of flight (TOF) determines the mass of each product. SpectroTYPER-RT software then converts mass data into genotypes, ensuring accurate SNP detection.

### 2.6. MassARRAY iPLEX Gold Assay Design

The assay design used SNP sequences from NCBI databases, with primers for PCR and single-base extension (SBE) created using MassARRAY software (v3.1). Multiplex primers were validated for uniqueness using BLAST tool provided in the NCBI databases [38]. Supplementary Table S1 provides primer details for each SNP used in genotyping.

### 2.7. Statistical Analysis

Multiple statistical tools have been employed to ensure the validity of the results in assessing the association between AA and different gene polymorphisms. Data analysis was conducted using Statistical Package for Social Sciences (SPSS) version 26.0 (SPSS, Inc., Chicago, IL, USA). Hardy–Weinberg equilibrium was employed to test the distribution of the polymorphisms in both patients and controls using the SNPStats web tool (https://www.snpstats.net/start.htm, accessed on 1 December 2024). To assess the risk of developing AA in Jordanians, allele and genotype frequencies of the selected polymorphisms were calculated between controls and patients. The frequency distribution between the two groups was assessed using the chi-square (χ^2^) test, and significance was set at *p*-value < 0.05. To accurately assess the impact of repeated testing on a particular trait, the Li and Ji approach [39] was utilized to determine the actual number of SNPs. This approach uses a modification of a prior Nyholt (2004) technique [40]. The obtained adequate number of SNPs (21) is then used in a modified Bonferroni method to find a target alpha level (0.05/21) that will maintain a total significance level of 0.0024 or below.

## 3. Results

### 3.1. Clinical and Demographic Data of Participants

The patient group consisted of 107 males and 45 females with AA, with an age range of 13 to 67 years with a mean ± standard deviation (SD) of 33.9 ± 9.81 years. The 150-individual control group comprised 129 males and 21 females (age range = 17–64 years, mean age ± SD = 33.9 ± 9.81 years). Table 1 shows the complete clinical and demographic data of the patients.

### 3.2. Hardy–Weinberg Equilibrium

The Hardy–Weinberg equilibrium (HWE) test was performed to evaluate the distribution of the selected gene polymorphisms in both the patient and control groups of the Jordanian population. The results demonstrated that all of the analyzed polymorphisms were in equilibrium (*p* > 0.05) (Table 2), indicating their normal distribution. Therefore, these polymorphisms were appropriate for inclusion in further analyses.

### 3.3. Genotyping and Association Analysis

Allele and genotype frequency analyses were conducted to assess the potential association between the selected gene polymorphisms and alopecia areata (AA) susceptibility in the Jordanian population. The results demonstrated no statistically significant differences in the allele or genotype distributions between the AA patients and controls, indicating no evidence of an association between the analyzed polymorphisms and AA susceptibility (Table 3).

## 4. Discussion

Alopecia is found to affect individuals across all age groups, with a generally higher prevalence observed in younger patients [41,42]. In our study, around 60% of the reported cases occurred in patients between 10 and 25 years of age, while in other populations, the disease occurs more frequently in patients between the ages of 21 and 40 [7,42,43,44]. Although the condition affects men and women equally, resulting in a stressful experience for both, the psychological consequences are typically considerably more distressing for affected women, given social beliefs regarding body image [45,46]. This situation might explain the more significant proportion of male patients (70.4%) who participated than females (29.6%). 

Most of the patients (90.13%) presented with patchy hair loss on the scalp or body, 3.28% presented with complete scalp alopecia, and the entire body was affected in 6.57% of the cases (Table 2). AA phenotypes can be subclassified into several forms, which vary in severity from mild patchy lesions to severe AT/AU [47]. Nevertheless, progression to severe forms is relatively rare, accounting for only 5% of AA cases [48]. Typically, the scalp is the most affected site in AA; other hair-bearing areas, such as eyebrows, beards, axillary areas, and the pubic area, can also be involved [9,15,49]. In agreement with preliminary reports, the patchy form of AA was the most common type among our patients, predominantly involving the scalp (60.5%). As a systemic disease, AA can also affect the nails with a wide range of dystrophies, including beau lines, pits, onychomadesis, ridges, and trachonychia [50,51]. Nail abnormalities are one of the poor prognosis indicators of AA that may occur during hair loss [6,50]. Previous studies have shown nail abnormalities in 30–67% of patients [3,9,15]. However, they were reported in 7.3% of the cases in this study. However, nail involvement is uncommon but usually associated with severe alopecia [52]. This is broadly consistent with our findings, since less than 10% of the patients in this study had either AT or AU.

Over time, numerous genetic factors have been identified in the literature as predisposing individuals to diseases of varying severity. However, these risk loci are likely attributed to individuality and may vary in other ethnic groups [53]. Therefore, this study analyzes a cohort of Jordanians regarding some of the previously reported candidate loci in comprehensive genetic association studies of AA (Table 3). One of the extensively studied variants associated with AA among different populations is PTPN22 rs2476601, a potent inhibitor of T-cell activation [54]. The rs2476601 SNP has been found to confer an increased risk of AA, particularly the mild patchy type, in Mexican [7], Egyptian [55], and Belgian/German populations [14]. In other studies, it has a stronger association with severe forms of the disease [1,56,57]. However, evidence from Iranian research [58] and the present study failed to confirm these findings with any forms of alopecia. Consistent with the whole-exome sequencing analysis of Asian patients with AU [15], HFE rs1799945, DEFB1 rs2738047, C20orf185 rs4911290, MICA rs9380254, CD96 rs2276872, DMBT1 rs2277244, CIITA rs78108426, CHIT1 rs2297950, and LTF rs1126477 also did not reach the significance level. Lactotransferrin (LTF) variant rs112647 was found to have a significant role in the physiology of ovarian cancer in Chinese Han patients [53]. However, the rs755622 variant of the macrophage migration inhibitory factor (MIF) did not show any association with severe AA, an early age of onset, or a positive family history in 768 patients with AA of Central European origin [59], comparable with this study’s findings. Nonetheless, this variation increases the MIF transcription levels, which may contribute to immune privilege protection, thus having a potential role in AA prevention.

PMS2 rs1805323 and HLA-DMB rs2071555 were two novel variants first reported in AA among the Asian population [15]. However, neither PMS2 nor HLA-DMB is associated with AA in the Jordanian cohort. The latter gene encodes HLA class II beta chain paralogues, such as HLA-DRB5, which plays a significant role in the human adaptive immune system [15]. A deficiency in the post-meiotic segregation increased 2 (PMS2) gene correlates strongly with impaired immunoglobulin class switch recombination; moreover, mutations in this gene are significantly associated with human cancer progression. Another HLA gene previously known as a genetic candidate for AA risk is HLA-A [60]. HLA-A rs60304108 is an additional locus recently reported for the development of AU [15]. In addition to GBP4 rs17130745, this locus is a monomorphic SNP in Jordanians, where rs17130745 is reported to have no association with AA in other populations [15]. Upon a meta-analysis of a genome-wide association study of AA susceptibility loci in a cohort of unrelated individuals (3253 patients and 7543 controls), ERBB3 rs2292239, RAET1L rs9479478, PRDX5 rs574087, and CLEC16A rs3862469 were identified as being associated with a risk of AA, in addition to two novel loci outside the major histocompatibility complex, including ACOXL/BCL2L11 rs3789129 and SH2B3(LNK)/ATXN2 rs653178 [14]. The variants above were further analyzed using three different analytic techniques to discover their associated etiological processes, in which these genes function in several immunological pathways [61]. Nevertheless, none of these genes were associated with AA in the present study. 

In the same cohort of this study, it was indicated previously that the A allele of the rs11073001 SNP in the *IL16* exon was more frequent in AA patients, with a significant difference between the patients and controls for the rs17875491 SNP in the *IL16* promoter region [62]. Furthermore, a significant distribution of TLR1 rs4833095, MASP2 rs2273346, and C11orf30 rs2155219 was observed [63]. The allele frequency of IL17RA rs879575 was higher among patients, while the AA genotype of IL31RA rs161704 was associated with AA development [64]. Additionally, CLEC4D rs4304840 variants, particularly in codominant and recessive models, were linked to AA susceptibility [65].

## 5. Limitations

This study has several limitations that should be acknowledged. The relatively small sample size, particularly for the alopecia totalis (AT) and alopecia universalis (AU) subgroups, may limit the statistical power to detect robust genetic associations specific to these severe forms of alopecia areata (AA). Additionally, while 21 SNPs were genotyped using the Sequenom MassARRAY platform, other potentially relevant genetic variants, epigenetic modifications, and environmental factors were not assessed, which may contribute to disease susceptibility. Furthermore, the study focused on genetic associations without incorporating environmental or immunological factors that may play a role in AA pathogenesis. Moreover, the case–control design does not establish causality. Future research should aim to increase the sample size, particularly for rarer AA subtypes, to enhance the statistical power and subgroup analyses. Additionally, genome-wide association studies (GWASs) or next-generation sequencing (NGS) approaches could help identify additional susceptibility loci. Integrating functional studies, epigenetic analyses, and immune profiling may provide deeper insights into the underlying mechanisms of AA pathogenesis. Longitudinal studies are also warranted to explore the causal relationships between genetic variants and disease progression.

## 6. Conclusions

The present study found no significant association between the analyzed SNPs and alopecia areata (AA) susceptibility in the Jordanian cohort. Our findings support or contradict reported results from other populations, suggesting that the disease can vary between ethnicities and may have a complex immunopathogenesis with multi-players. Regardless of the genetic predisposition, epigenetic, stress, and environmental factors will likely trigger pathologic immune responses. For instance, micronutrients have emerged as a possible cause of autoimmune diseases, including AA, where serum vitamin D, zinc, and folate levels tend to be lower in AA patients than in controls [20]. Clinically, these findings underscore the importance of incorporating not only genetic screening but also the evaluation of nutritional deficiencies, stress management, and immune dysregulation in the treatment of patients with alopecia areata (AA). Future research should focus on integrating genetic, epigenetic, and immune factors to develop personalized, multifactorial treatment strategies for AA.

## Figures and Tables

**Table 1:** Demographic and clinical data of AA patients.

Characteristics	Category	Patients
Age (years)	mean ± SD	31.144 ± 12.41
Onset age (years)	mean ± SD	27.328 ± 12.57
	<30 years	87, 57.3%
	≥30 years	65, 29.6%
Sex (n, %)	Males	107, 70.4%
	Females	45, 29.6%
Clinical subtypes (n, %)	PA	137, 90.13%
	AT	5, 3.28%
	AU	10, 6.57%
Affected sites (n, %)	Scalp	92, 60.5%
	Face	35, 23.02%
	Scalp and face	8, 5.3%
	Other body parts	4, 2.63%
Nail abnormalities (n, %)	−ve	141, 92.7%
	+ve	11, 7.3%
Associated symptoms (n, %)	Symptomatic	48, 31.6%
	Asymptomatic	104, 68.4%

AA, alopecia areata; n, number; %, frequency. Age data are presented as mean ± SD, years. Other data are presented as n, %. PA, patchy alopecia; AT, alopecia totalis; AU, alopecia universalis.

**Table 2:** List of SNPs, their minor allele frequencies, and their HWE *p*-values.

Gene	Polymorphism	Control			Patients		
		MA	MAF	HWE *p*-Value	MA	MAF	HWE *p*-Value
*PMS2*	rs1805323	T	0.04	1	T	0.04	1
*PRDX5*	rs574087	G	0.27	0.84	G	0.28	0.84
*ATXN2*	rs653178	C	0.38	0.73	C	0.39	0.12
*MIF*	rs755622	C	0.18	0.78	C	0.24	0.07
*LTF*	rs1126477	T	0.36	1	T	0.28	1
*HFE*	rs1799945	G	0.13	1	G	0.12	0.69
*HLA-DMB*	rs2071555	T	0.04	1	T	0.04	1
*CD96*	rs2276872	C	0.01	1	C	0	1
*DMBT1*	rs2277244	T	0.06	1	T	0.06	1
*ERBB3*	rs2292239	T	0.24	0.65	T	0.29	0.85
*CHIT1*	rs2297950	T	0.27	1	T	0.27	1
*PTPN22*	rs2476601	A	0.02	1	A	0.01	1
*DEFB1*	rs2738047	T	0.01	1	T	0.01	1
*ACOXL*	rs3789129	C	0.18	0.16	C	0.17	1
*CLEC16A*	rs3862469	T	0.37	0.72	T	0.35	0.59
*C20orf185*	rs4911290	A	0.35	1	A	0.41	0.74
*MICA*	rs9380254	C	0	1	C	0.01	1
*RAET1L*	rs9479478	G	0.41	0.87	G	0.4	0.09
*CIITA*	rs78108426	A	0.02	1	A	0.02	1
*HLA-A*	rs60304108	Monomorphic					
*GBP4*	rs17130745	Monomorphic					

MA: minor allele. MAF: minor allele frequency. An SNP was considered normally distributed when the *p*-value was greater than 0.05.

**Table 3:** Frequencies of alleles and genotypes in candidate gene polymorphisms in AA patients and control individuals.

Gene	SNP	Allele/Genotype	Control (n = 150) (n, %)	AA Patients (n = 152) (n, %)	*p*-Value
*PMS2*	rs1805323	G	283, 96	292, 96	1
		T	13, 4	12, 4	
		GG	135, 91	140, 92	0.78
		GT	13, 9	12, 8	
*PRDX5*	rs574087	A	213, 73	218, 72	0.88
		G	79, 27	84, 28	
		AA	78, 53	79, 52	0.98
		AG	57, 39	60, 40	
		GG	11, 8	12, 8	
*ATXN2*	rs653178	T	180, 62	184, 61	0.14
		C	112, 38	118, 39	
		CC	20, 14	18, 12	0.68
		TC	72, 49	82, 54	
		TT	54, 37	51, 34	
*MIF*	rs755622	G	240, 82	230, 76	0.079
		C	52, 18	72, 24	
		CC	5, 3	13, 9	0.13
		GC	42, 29	46, 30	
		GG	99, 68	92, 61	
*LTF*	rs1126477	C	189, 64	218, 72	1
		T	107, 36	86, 28	
		CC	60, 41	78, 51	0.12
		CT	69, 47	62, 41	
		TT	19, 13	12, 8	
*HFE*	rs1799945	C	257, 87	269, 88	0.59
		G	37, 13	35, 12	
		CC	112, 76	118, 78	0.81
		CG	33, 22	33, 22	
		GG	2, 1	1, 1	
*HLA-DMB*	rs2071555	G	285, 96	292, 96	1
		T	11, 4	12, 4	
		GG	137, 93	140, 92	0.88
		GT	11, 7	12, 8	
*CD96*	rs2276872	G	292, 99	303, 100	1
		C	2, 1	1, 0	
		GC	2, 1	1, 1	0.54
		GG	145, 99	151, 99	
*DMBT1*	rs2277244	C	277, 94	283, 94	0.62
		T	19, 6	19, 6	
		CC	129, 87	132, 87	0.95
		CT	19, 13	19, 13	
*ERBB3*	rs2292239	G	225, 76	216, 71	0.66
		T	71, 24	88, 29	
		GG	84, 57	76, 50	0.35
		GT	57, 39	64, 42	
		TT	7, 5	12, 8	
*CHIT1*	rs2297950	C	210, 73	212, 73	1
		T	76, 27	80, 27	
		CC	77, 54	77, 53	0.97
		CT	56, 39	58, 40	
		TT	10, 7	11, 8	
*PTPN22*	rs2476601	G	289, 98	302, 99	1
		A	7, 2	2, 1	
		GA	7, 5	2, 1	0.075
		GG	141, 95	150, 99	
*DEFB1*	rs2738047	C	291, 99	300, 99	1
		T	3, 1	4, 1	
		CC	144, 98	148, 97	0.74
		CT	3, 2	4, 3	
*ACOXL*	rs3789129	A	244, 82	253, 83	0.41
		C	52, 18	51, 17	
		AA	103, 70	105, 69	0.58
		AC	38, 26	43, 28	
		CC	7, 5	4, 3	
*CLEC16A*	rs3862469	C	183, 63	195, 65	0.9
		T	107, 37	107, 35	
		CC	59, 41	61, 40	0.67
		CT	65, 45	73, 48	
		TT	21, 14	17, 11	
*C20orf185*	rs4911290	G	191, 65	179, 59	0.71
		A	103, 35	125, 41	
		AA	18, 12	27, 18	0.3
		GA	67, 46	71, 47	
		GG	62, 42	54, 36	
*MICA*	rs9380254	G	293, 100	301, 99	1
		C	1, 0	3, 1	
		GC	1, 1	3, 2	0.32
		GG	146, 99	149, 98	
*RAET1L*	rs9479478	T	172, 59	183, 60	0.19
		G	122, 41	121, 40	
		GG	26, 18	29, 19	0.55
		TG	70, 48	63, 41	
		TT	51, 35	60, 39	
*CIITA*	rs78108426	C	291, 98	299, 98	1
		A	5, 2	5, 2	
		CA	5, 3	5, 3	0.97
		CC	143, 97	147, 97	
*HLA-A*	rs60304108	G	296, 100	304, 100	Monomorphic SNP
*GBP4*	rs17130745	T	294, 100	304, 100	Monomorphic SNP

AA, alopecia areata; n, number; %, frequency. *p*-values < 0.0023 (0.05/# of SNPs, 0.05/21 = 0.0023 after applying multiple comparisons) are considered significant.

## Data Availability

The data used to support the findings of this study are available from the corresponding author upon request.

## Supplementary Materials

The following supporting information can be downloaded at: https://www.mdpi.com/article/10.3390/medicina61030409/s1, Table S1: The primers (forward, reverse, and extension) used in SNP genotyping.
