# Supplementary material for: Investigating the Genetic Association of Selected Candidate Loci with Alopecia Areata Susceptibility in Jordanian Patients
Source: Medicina (Kaunas). 2025 Feb 26;61(3):409. doi: 10.3390/medicina61030409 (PMC11943738; doi:10.3390/medicina61030409)
Supplement: Supplementary file 1 [file medicina-61-00409-s001.zip › medicina-3464548-supplementary.pdf]

Supplementary Table S1: The primers (forward, reverse, and extension) used in SNP genotyping.

Gene	SNP_ID	PCR Primer 1	PCR Primer 2	Extension Primer
PMS2	rs1805323	ACGTTGGATGCTCAGAATCCACGGAAGTGC	ACGTTGGATGCAGTGAGTTCCAGTCACGG	CCACCTCCGCTCTGTCC
PRDX5	rs574087	ACGTTGGATGACAAGCTTTGCAGAGTGGAC	ACGTTGGATGACCTGACCTAACCCTTCCTG	GAGTGGACGGATGCTTG
ATXN2	rs653178	ACGTTGGATGAGGATCTTAGGGCTCTAGTC	ACGTTGGATGGTGCCTAATGGCTGCAATAT	GAGTATGAAGATGTCCTATGTCATG
MIF	rs755622	ACGTTGGATGCGATTTCTAGCCGCCAAGTG	ACGTTGGATGTCCAGCAACCGCCGCTAAG	GCCAAGTGGAGAACAG
LTF	rs1126477	ACGTTGGATGAATCCTTACTCCTTGGCCCC	ACGTTGGATGATTTTGTGGCCTCGGGTTG	TGAGTGTTCAGTGGTGC
HFE	rs1799945	ACGTTGGATGTGGAAACCCATGGAGTTCGG	ACGTTGGATGGTTTGAAGCTTTGGGCTACG	CCACCCCACACGGCGACTCTCAT
HLA-DMB	rs2071555	ACGTTGGATGAAGTGACTATCACGTGGAGG	ACGTTGGATGTATGTCCAGTCTCCATTGGG	CCTTTCATGCCTCACAGCAGTG
CD96	rs2276872	ACGTTGGATGCTATCGTATGGTTGCTGTTC	ACGTTGGATGCAAACCAATCAGAGATTCAC	CTTCGCTGTTCCATTCATCTG
DMBT1	rs2277244	ACGTTGGATGTGAGCAGATGACACCAGCGT	ACGTTGGATGTCAGGACACGAGTCTTACCT	TCCCCCAGCGTCTTCACTAT
ERBB3	rs2292239	ACGTTGGATGGCTATCACCCTTACTTCTGC	ACGTTGGATGACCCTAGATCCCTTAAGTGC	GTGAAGAGACTTTTGAATCTA
CHIT1	rs2297950	ACGTTGGATGATCCAGGAGCTTTACCACAC	ACGTTGGATGTGAAGACCCTGTTAGCCATC	CAACTAACTTCTGAGTGC
PTPN22	rs2476601	ACGTTGGATGAGATGATGAAATCCCCCCTC	ACGTTGGATGACTGAACTGTACTCACCAGC	GCCACCCTCCACTTCCTGTA
DEFB1	rs2738047	ACGTTGGATGGGTGGTAACTTTCTCACAGG	ACGTTGGATGGCAGAATAGAGACATTGCCC	AAGAAGATCTGATCATTACAATTGC
ACOXL	rs3789129	ACGTTGGATGTTTCTGTGTCAATGCTCAGG	ACGTTGGATGGGAATGTTATTAATTTGGCCC	CCCACTTTCAAATACAAATCT
CLEC16A	rs3862469	ACGTTGGATGAGATTTTGGCAGGACCGTAG	ACGTTGGATGTAACAGGGAGGTAAGACTGC	AGGCTCCAAATTGCTGA
C20orf185	rs4911290	ACGTTGGATGATGAGAGGCAATGTGGCCAG	ACGTTGGATGGCTTATAGGGTCGTTCTCGC	GCCAGAGAGAATTCCA
MICA	rs9380254	ACGTTGGATGATTTCCTCTTCCCCAGAGCC	ACGTTGGATGAACCCTGACTGCACAGATCC	GTAGAGCCCCACAGTCTTC
RAET1L	rs9479478	ACGTTGGATGCCAAGGGACATTGCAAAAGC	ACGTTGGATGTGCTGTCAGTTACCTGCGAG	ACATTGCAAAAGCTAGCTTA
CIITA	rs78108426	ACGTTGGATGCGGTGGGTGTTGGACTAAG	ACGTTGGATGAAAGTACCCTACAGGAGGAC	GGTTGGACTAAGCCTTTG
HLA-A	rs60304108	ACGTTGGATGCACTTCTGGAAGGTTCCATC	ACGTTGGATGTTCTACCCTGCGGAGATCAC	GGCACGAGCTCCGTGTC
GBP4	rs17130745	ACGTTGGATGGAATACTCCTAAGTCTTTTGC	ACGTTGGATGAAGCTGCTGCTTAGAAGCAC	CGATATGCTTTCTAGAGTAACCCT
